# A hybrid stacked ensemble and Kernel SHAP-based model for intelligent cardiotocography classification and interpretability

**DOI:** 10.1186/s12911-023-02378-y

**Published:** 2023-11-28

**Authors:** Junyuan Feng, Jincheng Liang, Zihan Qiang, Yuexing Hao, Xia Li, Li Li, Qinqun Chen, Guiqing Liu, Hang Wei

**Affiliations:** 1 School of Medical Information Engineering, Guangzhou University of Chinese Medicine, Guangzhou, China; 2 School of The Fifth Clinical Medical School, Guangzhou University of Chinese Medicine, Guangzhou, China; 3 Department of Human Centered Design, Cornell University, Ithaca, NY USA; 4 Third Affiliated Hospital, Guangzhou University of Chinese Medicine, Guangzhou, China; 5 Tianhe District People’s Hospital, First Affiliated Hospital of Jinan University, Guangzhou, China; 6 Guangzhou Sunray Medical Apparatus Co. Ltd, Guangzhou, China; 7 First Affiliated Hospital, Guangzhou University of Chinese Medicine, Guangzhou, China; 8 Intelligent Chinese Medicine Research Institute, Guangzhou University of Chinese Medicine, Guangzhou, China

**Keywords:** Cardiotocography, Fetal monitoring, Machine learning, Stacked ensemble, Kernel SHAP

## Abstract

**Background:**

Intelligent cardiotocography (CTG) classification can assist obstetricians in evaluating fetal health. However, high classification performance is often achieved by complex machine learning (ML)-based models, which causes interpretability concerns. The trade-off between accuracy and interpretability makes it challenging for most existing ML-based CTG classification models to popularize in prenatal clinical applications.

**Methods:**

Aiming to improve CTG classification performance and prediction interpretability, a hybrid model was proposed using a stacked ensemble strategy with mixed features and Kernel SHapley Additive exPlanations (SHAP) framework. Firstly, the stacked ensemble classifier was established by employing support vector machines (SVM), extreme gradient boosting (XGB), and random forests (RF) as base learners, and backpropagation (BP) as a meta learner whose input was mixed with the CTG features and the probability value of each category output by base learners. Then, the public and private CTG datasets were used to verify the discriminative performance. Furthermore, Kernel SHAP was applied to estimate the contribution values of features and their relationships to the fetal states.

**Results:**

For intelligent CTG classification using 10-fold cross-validation, the accuracy and average F1 score were 0.9539 and 0.9249 in the public dataset, respectively; and those were 0.9201 and 0.8926 in the private dataset, respectively. For interpretability, the explanation results indicated that accelerations (AC) and the percentage of time with abnormal short-term variability (ASTV) were the key determinants. Specifically, the probability of abnormality increased and that of the normal state decreased as the value of ASTV grew. In addition, the likelihood of the normal status rose with the increase of AC.

**Conclusions:**

The proposed model has high classification performance and reasonable interpretability for intelligent fetal monitoring.

## Background

Cardiotocography (CTG) is a tool for the judgment of fetal distress. It was introduced into fetal monitoring in the late 1960s and is still commonly utilized today due to its low cost and non-invasiveness [1]. CTG can monitor the changes in fetal heart rate (FHR) and the link to uterine contractions (UC). However, CTG is interpreted by obstetricians, whose inconsistency, subjectivity, and inexperience may possibly cause the current growth in the misdiagnosed rate [2]. Therefore, it’s essential to develop automated CTG classification models to assist obstetricians.

Artificial intelligence has exploded in the medical industry with the emergence of digital medical data and machine learning technologies. Several researchers have introduced machine learning (ML)-based models for intelligent CTG monitoring studies in SisPorto 2.0 Portugal by Ayresde et al. [3]. Das et al. applied a fuzzy-rule-based method to identify the fetus status [4]. Afridi et al. employed a correlation-based feature selection technique over the dataset to remove the unnecessary attributes and used Naïve Bayes to classify CTG data. The results revealed that the Naïve Bayesian classifier achieved an accuracy of 0.8306 [5]. Piri et al. explored fetal health status using an association-based classification approach, and the test findings showed that the associative classifier model created had an accuracy of 0.84 after feature selection [6]. Chen et al. established the deep forest classifier to solve the imbalanced data problems and improve fetal abnormality detection accuracy, eventually obtaining an accuracy of 0.9507 [7].

In general, the accuracy rates of the mostly existing CTG classification studies are above 80% [5–15]. However, it is challenging to trade off the performance and interpretability in these studies. On the one hand, simple algorithms, such as Baïve Bayes and decision tree, will sacrifice performance and result in serious bias problems. Still, the principles of their predictions are explainable. On the other hand, complex algorithms with high accuracy performance, such as ensemble and deep learning algorithms, are tough to interpret. For a practical model of intelligent fetal monitoring, the emphasis is not only on the predictive performance but also on the post hoc explanations.

In this study, a hybrid model was proposed to meet the challenge of the trade-off between performance and interpretability. On the intelligent CTG classification task, we established a stacked ensemble classifier to leverage the capabilities of several high-performing algorithms and achieve classification results that outperform individual algorithms. After obtaining the prediction results, we employed the Kernel SHapley Additive exPlanations (SHAP) framework for interpretation. Kernel SHAP is a model-independent method capable of interpreting various ML-based algorithms [16]. Our main contribution consists of two parts: (1) performing a stacked ensemble strategy learning mixed features to improve CTG classification performance; (2) firstly applying Kernel SHAP framework to solve the interpretability problem for complex intelligent CTG classification models.

The rest of the paper is organized as follows: Methods section presents the CTG datasets and the overall methodologies. The corresponding results which validated the proposed model are presented in Results section. These findings are further analyzed in Discussion section. Finally, Conclusions section concludes the work.

## Methods

**Fig. 1:**
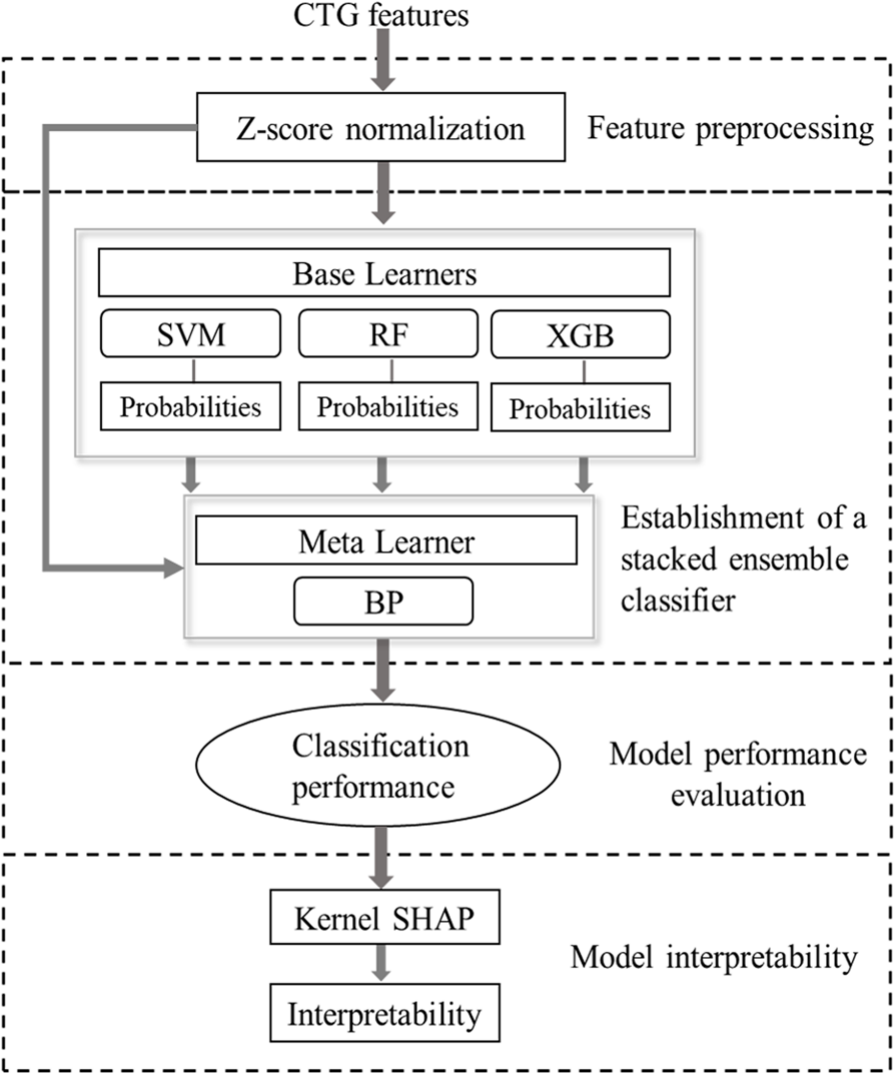
Design flow of the hybrid model. This figure illustrates the steps involved in the proposed model, including feature preprocessing, the establishment of a stacked ensemble classifier, evaluation of classification performance, and the analysis of model interpretability

This section describes the CTG datasets and the design flow of the hybrid model. The procedure is illustrated in Fig. 1 and consists of the following major steps: (1) CTG feature preprocessing, (2) stacked ensemble classifier establishment, (3) classification performance evaluation, and (4) model interpretability. In step 1, the CTG features were processed by zeros-mean normalization. Step 2 showed that a stacked ensemble strategy with mixed features was used to construct the proposed hybrid model in CTG classification part. In step 3, 10-fold cross-validation was used for evaluation. Finally, the predictive results were interpreted based on the Kernel SHAP framework in step 4.

### Datasets

The scientificity and validity of the approaches presented in this research were verified using the public and private CTG datasets.

#### The public CTG dataset

The public dataset is obtainable at the Machine Learning Repository of University of California [3]. It is one of the most authoritative datasets utilized for CTG retrospective studies. In the retrospective cohort, 2126 cardiotocograph signals with gestational weeks ranging from 29 to 42 weeks were processed, and the 21 structured features were calculated employing the SisPorto2.0 program. These signals were analyzed by three expert obstetricians based on Federation International of Gynecologie and Obstetrigue (FIGO) criteria, and each of them was given a consensus categorization label for fetal states (NSP, N=normal, S=suspicious, P=pathologic). In total, 1655, 295, and 176 cases are identified as normal, suspicious, and pathologic, respectively (Table 1).

**Table 1:** Attribute information of the public CTG dataset

Attribute	Description	Mean	Min	Max
LB	FHR baseline (beats per minute)	133.3	106	160
AC	number of accelerations per second	0.00	0	0.02
ASTV	percentage of time with abnormal short-term variability	47.0	12	87
ALTV	percentage of time with abnormal long-term variability	9.8	0	91
MLTV	mean value of long-term variability	8.2	0	50.7
MSTV	mean value of short-term variability	1.3	0.2	7
DP	number of prolonged decelerations per second	0.00	0	0.005
DS	number of severe decelerations per second	0.00	0	0.001
DL	number of light decelerations per second	0.00	0	0.02
Min	minimum of FHR histogram	93.6	50	159
Max	maximum of FHR histogram	164.0	122	238
Mode	histogram mode	137.5	60	187
Mean	histogram mean	134.6	73	182
Median	histogram median	138.1	77	186
Nmax	number of histogram peaks	4.1	0	18
Nzeros	number of histogram zeros	0.3	0	10
Width	width of FHR histogram	70.4	3	180
Variance	histogram variance	18.8	0	269
UC	number of uterine contractions per second	0.00	0	0.02
FM	number of fetal movements per second	0.01	0	0.5
Tendency	histogram tendency	Left-asymmetric=165 ; Symmetric=1115 ; Right-asymmetric=846		
Label	NSP (N : Normal; S : Suspicious; P : Pathologic)	N = 1655 ; S = 295 ; P = 176		

#### The private CTG dataset

In this retrospective study, 23,500 fetal morning cases from pregnant women with 28-42 weeks gestational ages were acquired in the collaborating hospitals between 2016 and 2018. Each fetal monitoring case contains fetal heart rate signal, uterine contraction signal, and clinical data of pregnant women. The signals were sampled at 1.25 Hz with SRF618A pro fetal monitor. The collection process of these private CTG data was approved by the local ethics committee and participants’ informed consent. Following the interpretation by three obstetricians as normal, suspicious, and pathologic statuses according to the ninth edition of the Chinese Obstetrics and Gynecology Fetal Monitoring Guidelines [17], 16,355 cases with consistent interpreting results were included in the private dataset for research. Therein, 11,998, 4,326, and 31 instances were judged as normal, suspicious, and pathologic, respectively. The pathologic class was relatively rare in the real-world clinic data and could not satisfy the classification criteria for experiments. Hence, the pathologic and the suspicious cases were merged into the abnormal category. Considering the clinical knowledge and remote fetal monitoring demands, 26 features (24 CTG features and two pregnant women’s characteristics) were employed as the classification inputs (Table 2). These 24 CTG features, such as FHR baseline and accelerations, were extracted from the CTG signals using SRF618A pro fetal monitor.

**Table 2:** Attribute information of the private CTG dataset

Attribute	Description	Mean	Min	Max
LB	FHR baseline (bpm)	141.52	112	181
AC	number of accelerations	4.09	0	22
AA	acceleration amplitude	18.56	0	75
AD	duration of accelerations	14.81	0	41
STV	short term variability	7.78	1.48	27.48
SD	number of severe decelerations	0.00	0	1
VA	variability of FHR	15.13	4	48
VD	number of variable decelerations	0.02	0	5
LD1	number of light decelerations	0.00	0	1
LD2	number of late decelerations	0.04	0	7
ED	number of early decelerations	0.00	0	1
DVHF	duration of variability in high frequency	11.00	0	50
DAD	duration of accelerations and decelerations	10.42	1	39
DVD	duration of variation decelerations	0.91	0	106
DUC	duration of uterine contractions	33.31	0	142
DVLF	duration of variability in low frequency	0.68	0	20
DC	number of decelerations	0.03	0	5
DL	data loss (%)	0.89	0	39
PD	number of prolonged decelerations	0.00	0	1
PV	periodic variation of FHR	3.88	1	11
TUC	interval time of uterine contractions	137.8	0	2559
IUC	intensity of uterine contractions	30.32	0	118
UC	number of uterine contractions	1.04	0	10
FM	number of fetal movements	8.4	0	49
GA	gestational age (week)	36.3	28	46
AGE	age of pregnant woman (year)	27.2	24	54
Label	Normal; Abnormal	Normal fetal state= 11,998 ; Abnormal fetal state= 4,357		

### Feature preprocessing

Each feature in the CTG datasets has different value ranges and units. Hence, numerical characteristics were automatically processed using zeros-mean normalization. The equation is as follows:

1 $$\begin{aligned} x*_{=} \frac{x-\mu }{\sigma } \end{aligned}$$

### A stacked ensemble strategy learning mixed features

The stacking algorithm is a powerful hierarchical ensemble learning algorithm. It employs a meta-learning algorithm to learn how to integrate the predictions from several base-learning algorithms, allowing it to tap into various high-performing classifiers to achieve results that outperform any single classifier.

**Fig. 2:**
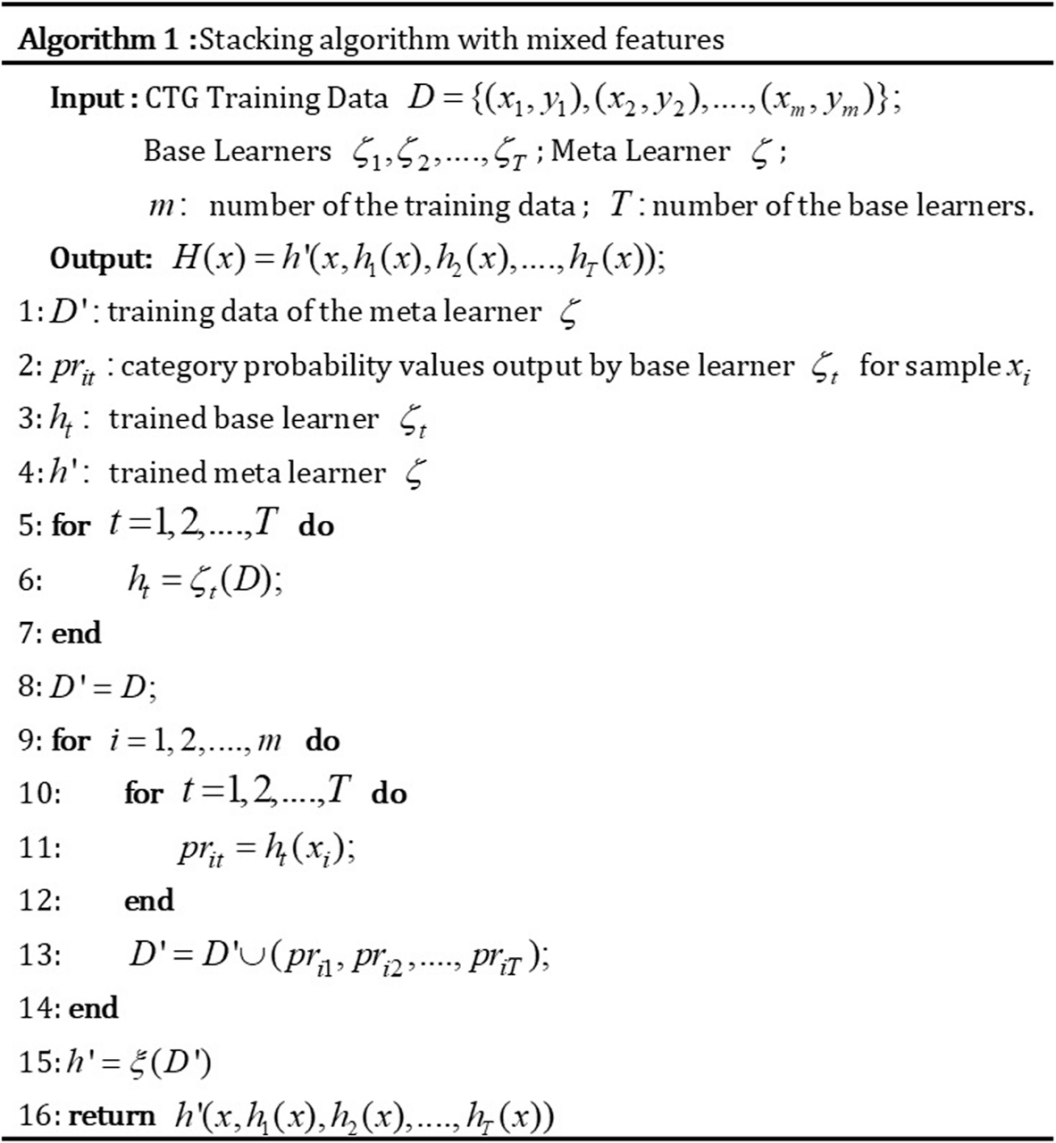
Stacking algorithm with mixed features. This figure describes how category probability values from base learners and CTG features are combined as input for the meta learner in the stacking ensemble strategy

During the stacking, the probability value of each category was implemented as the output by the base learners instead of their category labels for extracting more detailed CTG information. Then, these probability values and the CTG features were mixed as the input to the meta learner. In the proposed stacked ensemble strategy, the final predictions of the meta learner depend not only on the deep-level features extracted by the base learners but also on the original features of CTG data. The stacking algorithm with mixed features is shown in Fig. 2.

In the establishment, support vector machines (SVM), extreme gradient boosting (XGB), and random forests (RF) were utilized as base learners, and backpropagation (BP) was used as the meta learner. The selection of base learners is based on their complementary strengths, addressing specific challenges encountered in CTG classification strategically. SVM excels in solving nonlinear problems, RF effectively handles imbalanced datasets, while XGBoost mitigates high bias issues through boosting. The meta learner plays a crucial role in integrating the predictions from the base learners and CTG features to make the final ensemble prediction. Backpropagation (BP) is chosen as the meta-learner for its ability to perform nonlinear mapping. This allows BP to effectively transform the predictions from the base learners, resulting in more accurate and robust ensemble predictions.

### Evaluation metrics

The following metrics were utilized to measure the performance of the classification results: accuracy, precision, recall (sensitivity), and the F1 score.

Accuracy is computed as the ratio of the sum of true positive (TP) and true negative (TN) predictions to the total number of instances:

2 $$\begin{aligned} \text {Accuracy} = \frac{TP + TN}{TP + FP + TN + FN} \end{aligned}$$

Precision measures the ratio of true positive predictions to the sum of true positive and false positive (FP) predictions:

3 $$\begin{aligned} \text {Precision} = \frac{TP}{TP + FP} \end{aligned}$$

Recall (or Sensitivity) measures the ratio of true positive predictions to the sum of true positive and false negative (FN) predictions:

4 $$\begin{aligned} \text {Recall (Sensitivity)} = \frac{TP}{TP + FN} \end{aligned}$$

The F1 score is determined as the harmonic mean of precision and recall, providing a balanced evaluation under the imbalanced CTG classification task:

5 $$\begin{aligned} \text {F1 score} = \frac{2 \times \text {Precision} \times \text {Recall}}{\text {Precision} + \text {Recall}} \end{aligned}$$

### Kernel SHAP-based interpretability method

Kernel SHAP (SHapley Additive exPlanations) is a method that employs a specialized weighted linear regression function to compute the Shapley values, which estimates the contribution of each feature [16]. In the present study, Kernel SHAP was utilized to interpret the predictions made by the stacked ensemble classifier. The linear regression function g is defined as below:

6 $$\begin{aligned} g(z^{'} ) = \emptyset _{0} + {\textstyle \sum _{i=1}^{M}\emptyset _{i} z_{i}^{'} } , z_{i}^{'}\in \left\{ 0,1 \right\} ^{M} \end{aligned}$$

Here g is the explanation function, and $z^{'}$ is a feature coalition (1 = feature present in coalition, 0 = feature absent in coalition). M is the number of the CTG features. $\emptyset _{i}$ is the Shapley value for each CTG feature. Kernel SHAP aims to minimize the loss function L as below:

7 $$\begin{aligned} L(f,g,\pi )=\sum _{z^{'}\in Z} \left[ f(h(z^{'}))-g(z^{'}) \right] ^{2} \pi (z^{'}) \end{aligned}$$

Here f represents the classification model to be explained and $h(z^{'})$ maps a feature coalition into a feature set on which the model can be assessed. $f (h(z^{'}))$ is used to calculate the effect of features in present and absent. $\pi (z^{'} )$ is the weight assigned to the coalition (formula (8)). $|z^{'} |$ is the number of non-zero elements in $z^{'}$.

8 $$\begin{aligned} \pi (z^{'}) = \frac{(M-1)}{(M \, choose|z^{'} |)|z^{'} |(M-|z^{'} |)} \end{aligned}$$

By fitting the explanation linear regression function g in formula (6), the Shapley value $\emptyset _{i}$ is ultimately calculated to interpret the contributions of CTG features.

## Results

### The internal CTG classification performance comparisons

In this section, 10-fold cross-validation was employed for model evaluation. The evaluation results from 10 iterations will be averaged to obtain the final evaluation scores, effectively reducing overfitting and provide more reliable performance metrics.

#### Comparison of different stacking strategies

Table 3 shows the accuracy of four different stacking ensemble strategies under the public and private datasets, where Strategy 4 is the proposed stacking strategy. For metal learners’ input with different stacking strategies, Strategy 1 used the category labels, Strategy 2 replaced the category labels with the category probability values of the three base learners, and Strategy 3 applied a mixture of the category labels and original CTG features, respectively. In Table 3, the result indicates that the proposed stacking strategy outperformed the others, which benefits from the mixture of CTG features and the probability value of each category of base learners.

**Table 3:** Accuracy of different stacking strategies

Strategies	the public dataset	the private dataset
Strategy 1	0.9430	0.9129
Strategy 2	0.9513	0.9054
Strategy 3	0.9429	0.9130
Strategy 4	0.9539	0.9201

#### The performance of the proposed stacked ensemble strategy

Here, six ML-based algorithms, including logistic regression (LR), naïve bayes (NB), support vector machines (SVM), backpropagation (BP), random forests (RF), and extreme gradient boosting (XGB) were selected as comparison models. The metrics and comparison models were shown in Table 4, it can be seen that The proposed strategy is significantly better compared to other algorithms. When compared to the best single model SVM, the recall and precision in the proposed stacking were 0.0546 and 0.0362 higher, respectively. Since the F1 score could balance precision and recall, it was used to evaluate the performance of the classification results under imbalanced CTG data. The score of the proposed stacking was 0.9249, which was among the greatest overall comparison.

**Table 4:** The experimental results in the public dataset

Classifiers	Accuracy	Precision	Recall	Average F1
LR	0.8881	0.8053	0.7729	0.7847
NB	0.7198	0.6616	0.7439	0.7003
SVM	0.9315	0.8992	0.8621	0.8782
BP	0.9210	0.8680	0.8393	0.8514
RF	0.8949	0.8743	0.7170	0.7718
XGB	0.9314	0.9070	0.8610	0.8794
Proposed strategy	0.9539	0.9354	0.9167	0.9249

From Table 5, it can be also seen that the classification results of the stacking integration obtained better performance than without the integration in the overall comparison under the private dataset. The findings validated the predictive ability of the proposed stacked ensemble classifier with mixed features in real-world CTG data.

**Table 5:** The experimental results in the private dataset

Classifiers	Accuracy	Precision	Recall	Average F1
LR	0.8936	0.8737	0.8403	0.8548
NB	0.8481	0.8257	0.7546	0.7792
SVM	0.9125	0.8903	0.8781	0.8839
BP	0.9137	0.8920	0.8797	0.8856
RF	0.9054	0.9063	0.8402	0.8663
XGB	0.9130	0.8947	0.8737	0.8834
Proposed strategy	0.9201	0.9056	0.8816	0.8926

### Kernel SHAP-based interpretability

The Shapley values quantify the impact of each feature on the model’s predictions. After computing the Shapley values, we utilize the SHAP summary plot and the SHAP force plot to display the interpretability of the stacked ensemble classifier’s predictions. The summary plot displays a summary of feature importance for all instances in the dataset, which provides a comprehensive view of how each feature contributes to the model’s predictions across the entire dataset. The force plot is another visualization tool for interpreting individual predictions of machine learning models.

#### Summary plot of public dataset

The SHAP summary plot provides a global understanding of feature importance and their impact on the model’s predictions across the entire dataset. On the plot, features are ranked in descending order of importance on the y-axis, with the most crucial features displayed at the top.

**Fig. 3:**
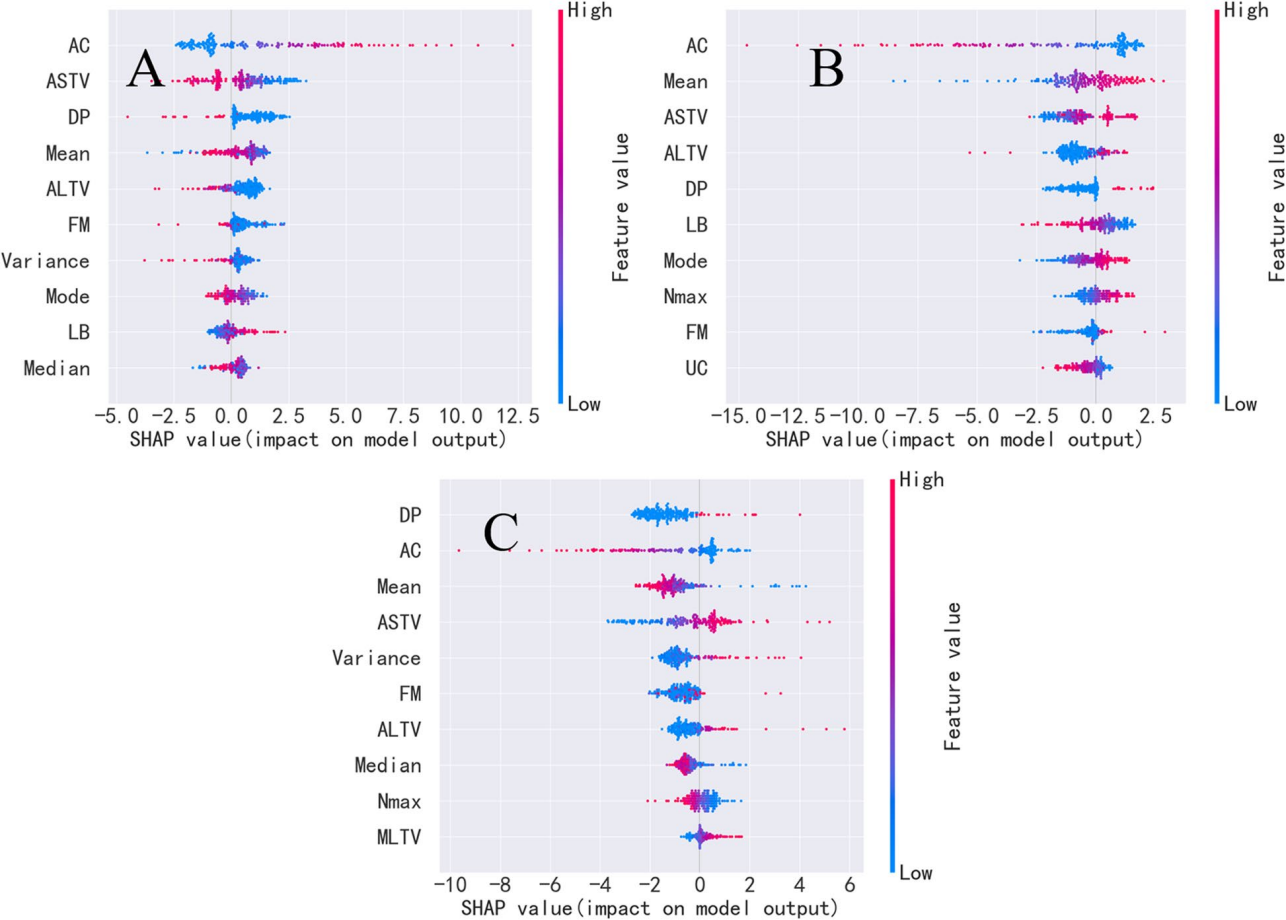
Summary plot of normal (**A**), suspect (**B**) and pathologic (**C**) category in the public dataset. This figure displays the top ten features contributing most to the prediction of fetal status for normal, suspicious, and pathological categories. Each point on the graph represents a Shapley value for a feature, with colors indicating the magnitude of the value

Figure 3 presents the summary plots for the normal, suspicious, and pathologic categories, showcasing the top ten features contributing to the fetal status predictions. Each point on the plot represents a Shapley value for a feature of an instance, with colors indicating the magnitude of the value. Red denotes high feature values, and blue represents low feature values. Shapley values provide contribution scores for CTG features in the predicted results, revealing their positive and negative influences on the model’s predictions. These summary plots offer valuable insights into the model’s behavior, facilitating the identification of the most influential features for each fetal status category.

It could be found that percentage of time with abnormal short-term variability (ASTV), mumber of accelerations per second (AC), histogram mean (Mean), and percentage of time with abnormal long-term variability (ALTV) most significantly contributed to the normal and suspicious category discrimination (Fig. 3A & B). In addition, ASTV, AC, Mean, and number of prolonged decelerations per second (DP) had the most impact on the pathologic category (Fig. 3C). Specifically, as the values of ASTV and DP increased and the values of AC and Mean decreased, the probability of pathologic risk grew.

#### Force plot of public dataset

The SHAP force plot provides insights into how specific features influence individual predictions, allowing obstetricians to understand why the model made a particular prediction for a given instance. In the plot, the base value represents the average prediction of the model calculated in a non-feature input condition of the explanation function. The final output *f(x)* for the instance is the sum of the base value and the contributions from each feature.

**Fig. 4:**
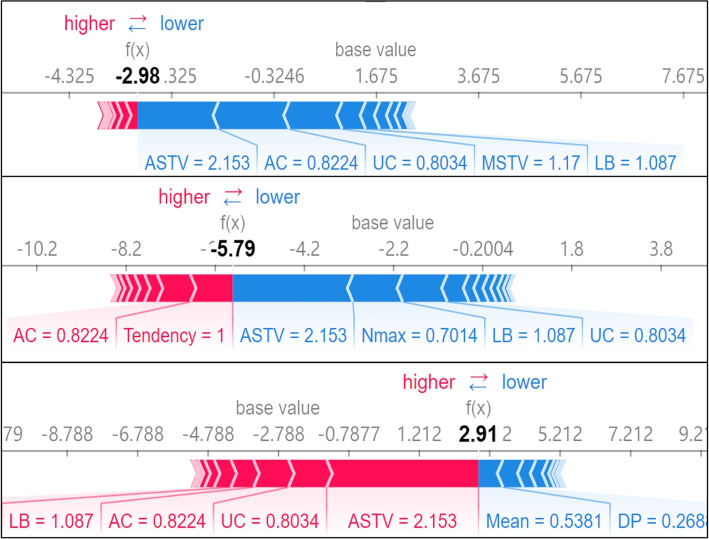
Interpretability of an individual case in the public dataset. This figure demonstrates how important features impact and explain predictions for normal, suspicious, and pathological categories for a given instance. Features driving the base value higher are highlighted in red, while those driving it down are highlighted in blue

Three force subplots in Fig. 4 showed that a sample randomly selected in the public dataset was interpreted as normal, suspicious, and pathologic status in sequence. The Shapley value of each feature pushes the base value to the ultimate output *f(x)*. The features that drive the output value higher are highlighted in red, while those that drive the value down are highlighted in blue. The classification model predicts the corresponding category outcome when a category *f(x)* surpasses the base value. As shown in the third subplot, the *f(x)* was 2.91, with main positive contributions from ASTV, AC, baseline beats per minute (LB), and number of uterine contractions per second (UC) and negative contributions from DP and Mean. Since the base value under the pathologic category interpretation was -2.788, the *f(x)* exceeded the base value and the sample was interpreted as pathologic status by the stacked ensemble classifier.

#### Summary plot of private dataset

**Fig. 5:**
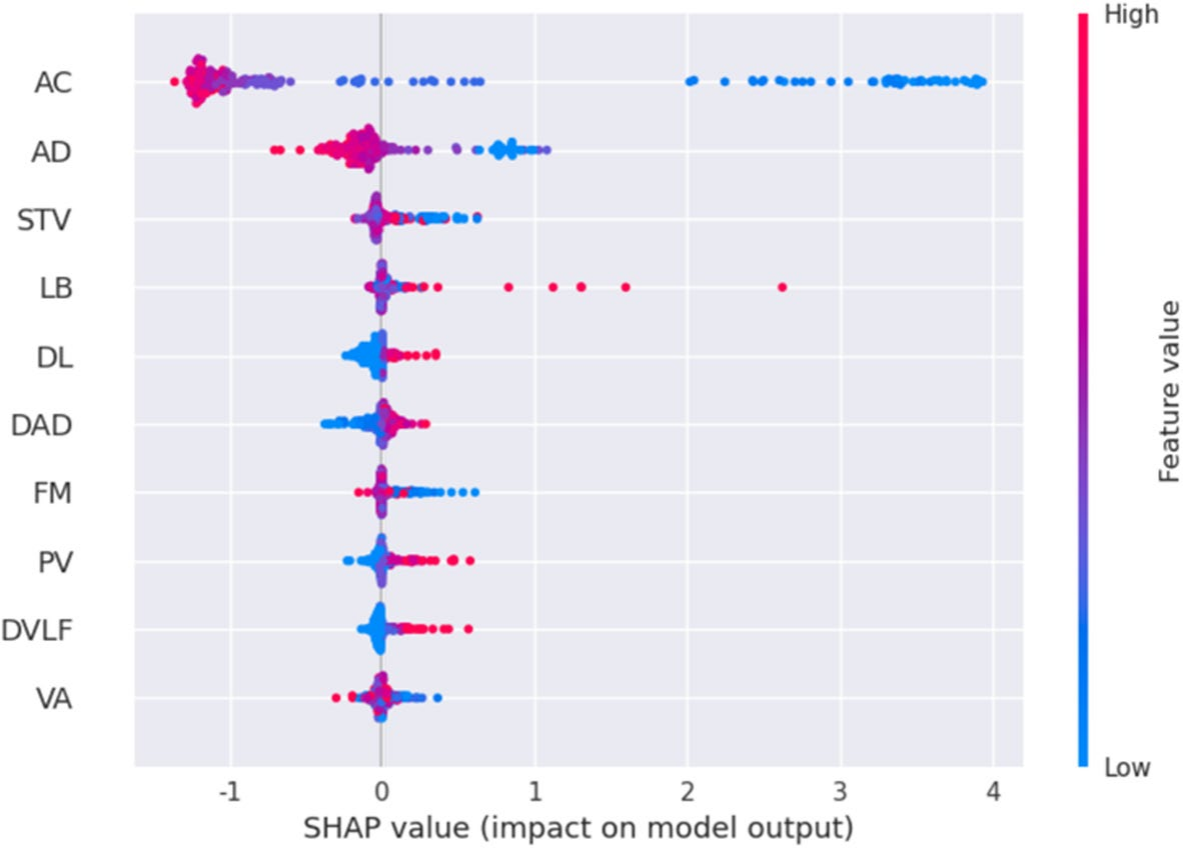
Summary plot of abnormal category in the private dataset. This figure displays the top ten most influential features affecting the model’s prediction when classifying fetal status as abnormal

Kernel SHAP was applied to the private data to verify explainability further. According to Fig. 5, AC, duration of accelerations (AD), and short term variability (STV) have the greatest influence on classifying fetal state as normal; the lower the value of these three characteristics, the more likely the fetal status would be judged as abnormal.

#### Force plot of private dataset

**Fig. 6:**
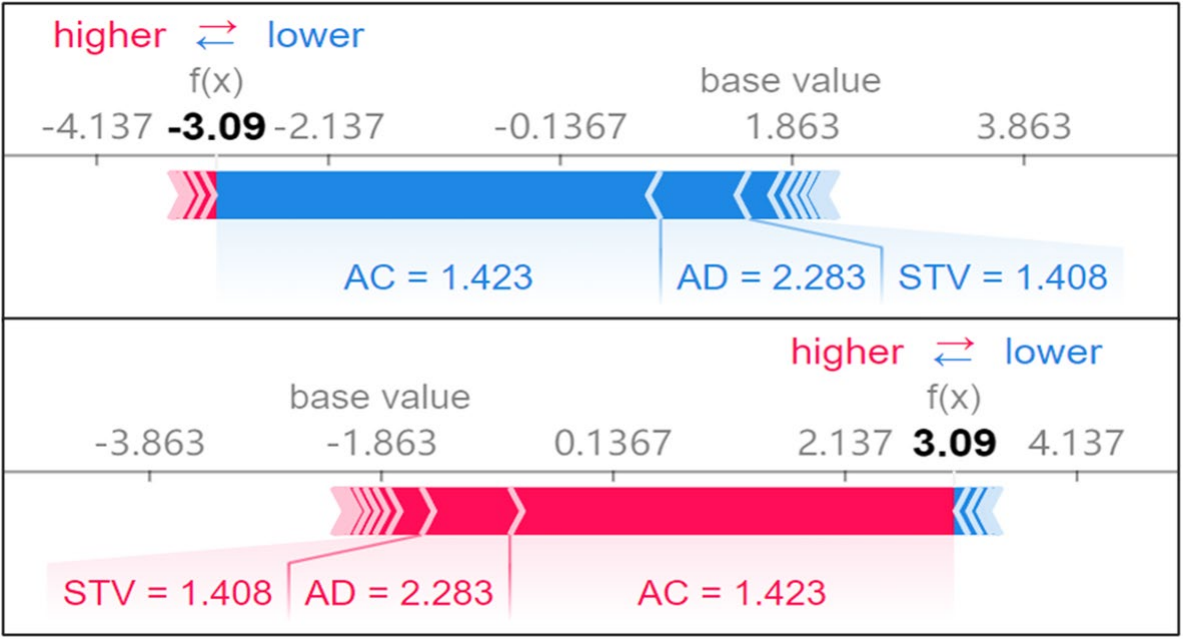
Interpretability of an individual case in the private dataset. This figure demonstrates how important features impact and explain predictions for the normal category in an individual instance

Two force subplots in Fig. 6 showed that a sample randomly selected in the private dataset was interpreted as normal and abnormal status in sequence. As shown in the second subplot, the *f(x)* was 3.09, with principal features of AC, STV, and AD playing positive roles in determining the classification results. Compared with the base value of -1.863 under the abnormal category interpretation, the *f(x)* was much higher. Thus, this sample was classified as an abnormal category.

## Discussion

### Comparison with existing ML based-models

In terms of internal comparisons (Table 3 & Table 4 & Table 5), the proposed strategy has a greater advantage. Additionally, to carry out an objective and comparative evaluation with the existing intelligent CTG classification models, we partition the public CTG dataset using the hold-out method. The public dataset was selected 70% randomly for training and the remaining 30% for testing. The confusion matrix of the stacked ensemble classifier constructed in this paper showed that the achieved accuracies were 0.9779, 0.8977, and 0.9434 in the normal, suspicious, and pathologic categories, respectively (Table 6). In particular, the misclassification rate between suspicious and normal was reduced considerably. To some extent, it could avoid the serious repercussions of postponing therapy owing to misjudging the suspicious class as the normal class.

**Table 6:** The experimental results in the public dataset

	Real		
	Normal	Suspicious	Pathologic
Predict			
Normal	0.9779	0.0795	0.0377
Suspicious	0.0161	0.8977	0.0189
Pathologic	0.0060	0.0227	0.9434

In Table 7, it can be seen that the performance of the proposed strategy has significantly improved compared with several existing CTG classification models [7–9], especially in detecting normal and suspicious categories. Compared with Probabilistic Neural Networ (PNN) [8], the F1 value of the suspicious class was further enhanced by 0.1596. In comparison with random forest [9], the F1 value of the suspicious class was further increased by 0.0717. Moreover, compared with Deep Forest (DF) [7], the suspicious F1 value grew by 0.0406. The results suggested that our classification model could significantly help prevent serious misdiagnosis problems, such as the misdiagnosis leading to postponing therapy.

**Table 7:** Comparison with several current CTG-based classifier in the public dataset

Classifiers	Categories	F1	Average F1	Accuracy
MLPNN [8]	Normal	0.9500	0.8131	0.9036
	Suspicious	0.6843		
	Pathologic	0.8050		
GRNN [8]	Normal	0.9570	0.8483	0.9186
	Suspicious	0.7392		
	Pathologic	0.8488		
PNN [8]	Normal	0.9591	0.8506	0.9214
	Suspicious	0.7381		
	Pathologic	0.8545		
Random Forest [9]	Normal	0.9730	0.8996	0.9480
	Suspicious	0.8260		
	Pathologic	0.9000		
DF [7]	Normal	0.9700	0.9201	0.9507
	Suspicious	0.8571		
	Pathologic	0.9333		
Proposed strategy	Normal	0.9779	0.9345	0.9639
	Suspicious	0.8977		
	Pathologic	0.9434		

### Model interpretability analysis of fetal status

As summarized in the interpretability results, the analysis suggested that AC and ASTV were key determinants impacting on fetal states in both the public and private datasets. Specifically, the probability of abnormality increased and that of the normal state decreased as the value of ASTV grew. In addition, the likelihood of the normal status rose with the increase of AC. According to the international fetal guidelines [18, 19], one of the most important conditions for normal state is that the AC rises at least 15 seconds when the increase of FHR baseline exceeds 15 beats per minute and this appeared more than twice in 15 minutes. Street discovered that STV was significantly associated with metabolic acidosis and a dead fetus in the uterus [20]. Huang concluded that AC and ASTV have more significant impacts on fetal status by combining the experimental results of Spearman correlation, data visualization, and association rules [21]. J.A. found that STV and long-term variability (LTV) are vital features in CTG by analyzing the correlation of STV, LTV [22], and decelerations (DC) [23] and heart rate variability (HRV) with fetal status. Santo conducted experiments to show that AC and DP are essential features [24]. These studies provide references for the validity of the interpretable results in this paper.

### Limitation

The CTG features for the experimental models in this study were extracted from the CTG signals, so there are still existing some non-negligible measurement errors. Despite deep learning (DL) can achieve end-to-end intelligent CTG classification, its interpretability could be even more complex. In the future, we plan to implement an intelligent CTG classification model based on DL and combined with Deep SHAP [16] to solve the problem of DL-based models that are difficult to interpret.

## Conclusions

As machine learning algorithms are increasingly being deployed in the healthcare domain, there is growing emphasis not only on predictive accuracy but also on techniques for explaining these black boxes. In this study, we presented a hybrid model to meet the challenge of the trade-off between performance and model interpretability. Both the public and private datasets were used to verify the model’s operability and applicability. The experimental results showed that the proposed model had superior classification performance, which is crucial for assisting obstetricians in assessing fetal health. Moreover, it enables the post hoc explanations of predictive results. Specifically, the contributions of different features in predicting fetal states have been elaborated. Therefore, our approaches contribute to the prenatal clinical application and implementation of intelligent fetal monitoring.

## Data Availability

The public CTG data used in this work is available from https://archive.ics.uci.edu/ml/ datasets/cardiotocography. Due to privacy restrictions, the private CTG dataset obtained from the collaborating hospitals cannot be publicly available.

## Abbreviations

CTG: Cardiotocography
FHR: Fetal heart rate
UC: Uterine contractions
FIGO: Federation International of Gynecologie and Obstetrigue
NSP: Normal, suspicious and pathological cases
ML: Machine learning
SHAP: SHapley Additive exPlanations
LR: Logistic regression
NB: Naïve bayes
SVM: Support vector machines
BP: Backpropagation
RF: Random forests
XGB: Extreme gradient boosting
ASTV: Percentage of time with abnormal short-term variability
AC: Number of accelerations per second
Mean: Histogram mean
ALTV: Percentage of time with abnormal long-term variability
DP: Number of prolonged decelerations per second
LB: FHR baseline (beats per minute)
STV: Short term variability
AD: Duration of accelerations
LTV: Long-term variability
DC: Number of decelerations
HRV: Heart rate variability
MLPNN: Multilayer perceptron neural network
GRNN: Generalized Regression neural network
PNN: Probabilistic neural network
DF: Deep forest

## References

[1] Grivell RM, Alfrevic Z, Gyte GM, Devane D. Antenatal cardiotocography for fetal assessment. Cochrane Database Syst Rev. 2015;2015(9):CD007863. 10.1002/14651858.CD007863.pub4. 10.1002/14651858.CD007863.pub4 PMC6510058 26363287

[2] Georgieva A, Redman C, Papageorghiou AT (2017). Computerized data-driven interpretation of the intrapartum cardiotocogram: a cohort study. Acta Obstet Gynecol Scand..

[3] Dua D, Graff C. UCI Machine Learning Repository. Irvine: University of California, School of Information and Computer Science; 2019. http://archive.ics.uci.edu/ml.

[4] Das S, Obaidullah SM, Santosh KC (2020). Cardiotocograph-based labor stage classification from uterine contraction pressure during ante-partum and intra-partum period: a fuzzy theoretic approach. Health Inf Sci Syst..

[5] Afridi R, Iqbal Z, Khan M (2019). Fetal heart rate classification and comparative analysis using cardiotocography data and KNOWN classifiers. Int J Grid Distrib Comput (IJGDC)..

[6] Piri J, Mohapatra P. Exploring fetal health status using an association based classification approach. In: 2019 International Conference on Information Technology (ICIT). IEEE; 2019. pp. 166–71. 10.1109/ICIT48102.2019.00036.

[7] Chen Y, Guo A, Chen Q (2021). Intelligent classification of antepartum cardiotocography model based on deep forest. Biomed Signal Process Control..

[8] Yılmaz E (2016). Fetal state assessment from cardiotocogram data using artificial neural networks. J Med Biol Eng..

[9] Imran Molla MM, Jui JJ, Bari BS, et al. Cardiotocogram data classification using random forest based machine learning algorithm. In: Proceedings of the 11th National Technical Seminar on Unmanned System Technology 2019. Singapore: Springer; 2021. pp. 357–69. 10.1007/978-981-15-5281-6_25.

[10] Kadhim NJA, Abed JK. Enhancing the prediction accuracy for cardiotocography (CTG) using firefly algorithm and naive Bayesian classifier. In: IOP Conference Series: Materials Science and Engineering, vol 745, issue 1. IOP Publishing; 2020. p. 012101. 10.1088/1757-899X/745/1/012101.

[11] Chen J, Liu X, Wei H (2019). Imbalanced cardiotocography multi-classification for antenatal fetal monitoring using weighted random forest. Int Conf Smart Health..

[12] Georgoulas G, Karvelis P, Spilka J (2017). Investigating pH based evaluation of fetal heart rate (FHR) recordings. Health Technol..

[13] Li J, Huang L, Shen Z (2019). Automatic Classification of Fetal Heart Rate Based on Convolutional Neural Network. IEEE Internet Things..

[14] Cmert Z, Kocamaz AF. Fetal Hypoxia Detection Based on Deep Convolutional Neural Network with Transfer Learning Approach, vol 763. Cham: Springer; 2019. pp. 239–48. 10.1007/978-3-319-91186-1_25.

[15] Hoodbhoy Z, Noman M, Shafique A (2019). Use of machine learning algorithms for prediction of fetal risk using cardiotocographic data. Int J Appl Basic Med Res..

[16] Lundberg SM, Lee SI. A unified approach to interpreting model predictions. In: Proceedings of the 31st international conference on neural information processing systems, vol 2017. Long Beach: Neural Information Processing Systems Foundation, Inc. (NeurIPS); 2017. pp. 4768–77.

[17] Xie X, Kong B, Duan T. Gynecology and Obstetrics, vol. 2018. 9th ed. Beijing: People’s Health Publishing House; 2018. p. 54–6.

[18] Tomáš P, Krohova J, Dohnalek P, et al. Classification of cardiotocography records by random forest. In: 2013 36th International conference on telecommunications and signal processing (TSP), vol 2013. 2013. pp. 620–923. 10.1109/TSP.2013.6614010.

[19] Shah SAA, Aziz W, Arif M, et al. Decision trees based classification of cardiotocograms using bagging approach. In: 2015 13th international conference on frontiers of information technology (FIT), vol 2015. 2015. pp. 12–7. 10.1109/FIT.2015.14.

[20] Street P, Dawes GS, Moulden M, et al. Short-term variation in abnormal antenatal fetal heart rate records. Am J Obstet Gynecol. 1991;165(3):515–23. 10.1016/0002-9378(91)90277-X. 1892175

[21] Huang L, Jiang Z, Cai R (2021). Investigating the interpretability of fetal status assessment using antepartum cardiotocographic records. BMC Med Inform Decis Making..

[22] Pardey J, Moulden M, Redman CW (2002). A computer system for the numerical analysis of nonstress tests. Am J Obstet Gynecol..

[23] Bauer A (2006). Phase-rectified signal averaging detects quasi-periodicities in non-stationary data. Phys A..

[24] Santo S (2017). Agreement and accuracy using the FIGO, ACOG and NICE cardiotocography interpretation guidelines. Acta Obstet Gynecol Scand..

